# Fit of tooth‐supported zirconia single crowns—A systematic review of the literature

**DOI:** 10.1002/cre2.323

**Published:** 2020-09-03

**Authors:** Walaa Magdy Ahmed, Batoul Shariati, Arwa Z. Gazzaz, Mohammed E. Sayed, Ricardo M. Carvalho

**Affiliations:** ^1^ Faculty of Dentistry The University of British Columbia Vancouver British Columbia Canada; ^2^ Faculty of Dentistry King Abdulaziz University Jeddah Saudi Arabia; ^3^ College of Dentistry King Saud University Riyadh Saudi Arabia; ^4^ College of Dentistry Jazan University Jazan Saudi Arabia

**Keywords:** crown preparation, marginal gap, sintering, zirconium oxide

## Abstract

**Purpose:**

The purpose of this study is to systematically map all the factors that influence the fit and adaptation of zirconia crowns and/or copings.

**Materials and methods:**

The investigational strategy involved carrying out an electronic search between December 1, 2009 and September 1, 2019 through the Embase and Medline databases using Boolean operators to locate appropriate articles.

**Results:**

A total of 637 articles were discovered after the removal of duplicates, and 46 of these were selected for evaluation. Further, a quality assessment was performed using GRADE evaluation criteria.

**Conclusions:**

Shoulder finish line preparations had slightly better marginal fit compared to chamfer finish lines. Crowns obtained from digital impressions had comparable to superior marginal adaptation compared to conventional impressions. Increasing cement space showed to improve zirconia crown adaptation. Cementation and veneering zirconia frameworks found to increase the marginal and internal gaps. Limited information is available on the effect of the alteration of sintering time/Temperature and/or sintering techniques on the adaptation of zirconia crowns. Most of the selected studies had a moderate quality assessment evaluation. Future studies could investigate the chair‐side, ultra‐fast sintering effect on the marginal gap of zirconia crowns.

## 1 INTRODUCTION

Fit and adaptation are essential components in the long‐term success of a prosthetic crown (Abduo, Lyons, & Swain, 2010). Misfit of the crown prosthesis margin generates a potential space (marginal gap) between the restoration and the prepared tooth. Bacterial contamination can easily accumulate in the marginal gap and jeopardize the longevity of the treatment (Sailer, Makarov, Thoma, Zwahlen, & Pjetursson, 2015). Furthermore, adjustments of the prosthesis by grinding to achieve a proper fit can lead to stress concentrations; this may reduce the resistance to fracturing of the crown and, consequently, lead to clinical failure (Pak, Han, Lee, Kim, & Yang, 2010).

The importance of the marginal fit lies in the fact that the major causes of zirconia restorations failure are secondary caries and loss of retention (Sailer et al., 2015), which are factors closely related to the dissolution of the luting cement and deficiencies in marginal adaptation. Meanwhile, a minimum and uniform internal gap is also a desirable and important aspect of indirect restorations; this is because large and inhomogeneous internal gaps may negatively affect the retention or resistance of the restoration (Pedroche et al., 2016). It has been proposed that secondary caries (recurrent caries) are linked to microleakage at the tooth‐restoration interface and depend on the size of the marginal gap; however, this is not supported by either clinical studies or laboratory models (Nassar & Gonzalez‐Cabezas, 2011). In contrast, larger marginal gaps have been discovered to have a significant effect on the development of secondary caries in vitro and can be modified by fluoride applications (Nassar & Gonzalez‐Cabezas, 2011). Although a positive correlation has been found between the marginal gap size and the development of secondary caries (Totiam, Gonzalez‐Cabezas, Fontana, & Zero, 2007), no conclusive evidence has been found to relate the marginal gap size and/or placement (supra/subgingival) to secondary caries. Moreover, studies were not even able to find an association between marginal gap and microleakage (Cristian, Jeanette, Francisco, & Guillermo, 2016; Karl, Graef, Schubinski, & Taylor, 2012).

Routinely in clinical dentistry, a mirror and probe are used to evaluate the restoration margins. However, standardized criteria, such as those from the Modified US Public Health Service (USPHS) (Ryge, 1980) and California Dental Association (CDA) (California Dental Association, 1977), are used for the clinical evaluations of marginal dental prostheses in clinical studies to ensure quality. Owing to the fact that the USPHS and CDA criteria do not consider the presence of secondary caries, Zoellner, Bragger, Fellmann, and Gaengler (2000) proposed a clinical diagnostic index for secondary caries at the crown gingival margin that can assist in the management of secondary caries according to location and severity.

Among dental ceramics, zirconia has seen a marked increase in use in dentistry because of its white color and functional outcomes (Tabatabaian, 2018). In contemporary dentistry, the use of computer‐aided design/computer‐aided manufacturing (CAD/CAM) is the only technique for fabricating zirconia restorations. The most popular route for fabricating zirconia prostheses is to use partially sintered zirconia blanks using the soft‐milling technique, and the blank must be sintered to achieve the final density and maximum strength of the material. This sintering procedure is accompanied by a relatively high sintering shrinkage of approximately 20%–30% (Suttor, Bunke, Hoescheler, Hauptmann, & Hertlein, 2001). This sintering shrinkage is managed by instructing the software to mill an enlarged crown by an appropriate factor to compensate for this shrinkage after sintering (Besimo, Spielmann, & Rohner, 2001). Consequently, a certain degree of misfit is expected when trying‐in a crown on the original preparation.

It has been well established that misfit of prostheses may occur as a result of several different clinical and laboratory variables; these include deviation from the recommended guidelines of tooth preparation, inaccuracy of the impression taken, and/or firing cycles. Assessment of the post‐sintering dimensional change in zirconia in dentistry has been accomplished by means of measuring the marginal and internal fit of the prosthesis. Inadequate fitting of crowns is usually managed by accepting a larger cement line and/or by making post‐sintering bur adjustments to the crown to compensate for discrepancies. Post‐sintering adjustments can trigger tetragonal to monoclinic (t➔m) phase transformation, which can lead to potentially disastrous consequences for the prosthesis. The purpose of this study is to systematically review zirconia crowns and/or coping studies related to marginal and internal fit; further, this study is aimed to map all the factors that influenced the fit of zirconia crowns and/or copings.

## 2 MATERIALS AND METHODS

### 2.1 Focused question and search strategy

The focused question was determined according to the well‐established PICO strategy 2009. (1) Population: crown or coping prostheses fabricated in vitro. (2) Intervention: zirconium oxide material. (3) Comparison: N/A. (4) Outcome: marginal and internal adaptation. The focused question of the presented review was “Within the available in‐vitro studies on full coverage crown or coping prostheses, what are the factors affecting the marginal and internal adaptation of zirconium oxide crowns and/or copings?”

The search strategy involved carrying out an electronic search through Embase and Medline using Boolean operators to locate appropriate articles as described in Table 1. Systematic reviews and non‐English articles were excluded from the search. The electronic search was supplemented by manual searching for the last 8 years through the following journals: *Dental Materials*, *Journal of Oral Rehabilitation*, *Journal of Prosthetic Dentistry*, *Journal of Prosthodontics*, *International Journal of Prosthodontics*, *International Journal of Periodontics and Restorative Dentistry*, and *Quintessence International*. In addition, the references of the selected articles were reviewed for possible inclusion.

**TABLE 1:** Search strategy in MEDLINE applied for this review

Search	Literature search strategy	Results
**P**opulation	Crown* OR coping* OR exp crowns/	97,746
**I**ntervention	Zirconia OR zirconium OR yttrium‐stabilized tetragonal zirconia YTZP	3,662
**C**ontrol	N/A	N/A
**O**utcome	Marginal fit OR marginal adaptation OR internal fit OR internal adaptation OR accuracy OR discrepancy	1,581,267
**Total**	1 AND 2 AND 3 AND 4	**790**
**Exclusion**	Dental implants/or dental prosthesis, implant‐supported/, reviews	594
**Limit**	Limited to English language	574

### 2.2 Inclusion/exclusion criteria

The search inclusion criteria were: studies published between December 1, 2009 and September 1, 2019 and limited to in vitro studies published in peer‐reviewed journals, articles written in English, which contained all or part of the key words in their headings or abstract, articles assessing the marginal and/or internal adaptation on single crowns for tooth‐supported restorations. The search was restricted by excluding “implant‐supported prostheses” and review articles.

### 2.3 Selection criteria

**FIGURE 1:**
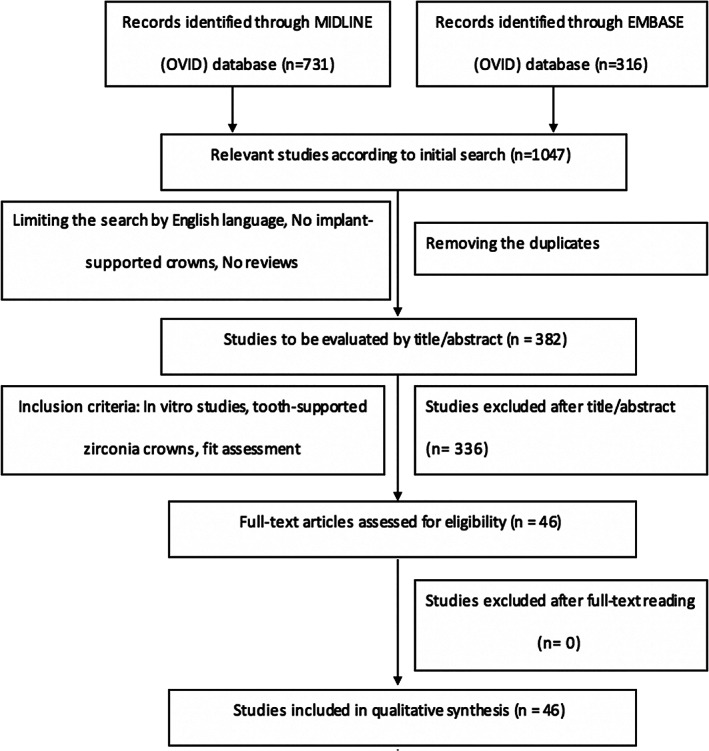
PRISMA flow diagram to identify the included studies in the review

The titles and abstracts of all articles were reviewed by two independent reviewers (W.A. and A.G.). Disagreement between the two reviewers was resolved by discussion. Upon identification of an abstract for possible inclusion, the full text of the article was reviewed and cross‐matched against the predefined inclusion criteria. Figure 1 illustrates the process of identifying the included articles in the review as a flow diagram. Table 2 describes the reasons for excluding studies.

**TABLE 2:** Descriptive table for excluded articles

Reason for exclusion	Results
Fracture and fatigue testing	65
Clinical studies and case reports	57
Different design, fixed partial denture (bridge), inlay, onlay, post, and core	38
Implant, implant‐supported restoration, implant abutment	34
Reviews	30
Bonding tests	25
Other all‐ceramic materials	22
Finite element analysis and stress distribution studies	15
Microleakage and thermocycling testing	14
Non‐relevant studies	14
Optical properties, translucency, color	10
Surface treatment of zirconia	6
Wear studies	3
Chipping studies	3
Total excluded articles	336

### 2.4 Data extraction

The following data were extracted from each article: type of fabrication system, factors tested that influenced the fit, sample size, type of finish line/preparation design, cement thickness, measurement method, number of measurements per sample, and marginal and internal gap measurements. Any variable that could not be extracted was scored as not reported (“nr”).

### 2.5 Quality assessment

The interobserver calibration was evaluated by Cohen's Kappa and the chosen cut‐off point was 80%. GRADE criteria were used to provide a framework for quality assessment of the selected studies (Balshem et al., 2011). The quality levels were High (H), Moderate (M), Low (L), and Very Low (VL). Quality reflects our confidence that the estimate of the effect is correct. GRADE separates the process of quality assessment of evidence from the process of formulating recommendations (Balshem et al., 2011). Decisions about the guideline developments relayed on more than just the quality of evidence.

## 3 RESULTS

The electronic search collectively revealed 1,047 articles, including 731 from Medline and 316 from Embase of which, after the removal of duplicates, 382 studies were processed for review based on an analysis of titles and abstracts. Searching manually and checking the references of the selected articles did not provide any further articles; therefore, only the articles from the electronic search were considered. The articles that did not meet the inclusion criteria were articles of in vivo studies, fixed dental prosthesis of two or more units, implant‐supported prostheses, testing other types of all‐ceramic restorations that were not zirconia, non‐English studies, non‐peer‐reviewed studies, and testing bond strength or scanning accuracy. Consequently, 46 articles were selected for quality assessment of marginal and internal fit of zirconia crowns. Most of them had moderate quality assessment, with a Kappa score of 0.8.

### 3.1 Factors affecting zirconia marginal fit

The factors that showed influence on the fit of zirconia crowns were the following: (N = number of papers)

- Use of different manufacturing systems (14), use of different zirconia materials (13);
- Comparisons between digital and conventional impression techniques (6);
- Effect of cementation (7);
- Effects of different finish line configurations (4), depth or curvature (2), width (1), cement space (1), and changing the occlusal preparation (1);
- Effect of veneering (5), using different veneering techniques (1);
- Influence of using different die materials with or without powder (1);
- Altering sintering protocols (2).

### 3.2 Methods used for measuring marginal and internal fit of zirconia crowns

Direct view technique was the method used in most (20) of the papers reviewed. The other methods were as follows: (N = number of papers)

- Sectioning after cementation (10);
- Replica technique (12), Digitalized replica technique (1);
- Triple‐scan optical protocol (2);
- Micro‐computed tomography (micro‐CT) (1);
- 3D coordinate measuring system (CNC Rapid) (1);
- Weight technique (1).

Assessing measuring points varied between studies even within each measuring technique, as shown in the summary table of included articles (Table 3). The same was true for the cement space selection. Owing to the high heterogeneity of the methodologies between the selected studies, it was difficult to draw a solid conclusion regarding the best methodology to evaluate the fitting accuracy of zirconia crowns. However, some studies confirmed the similarity between direct external and internal viewing techniques in measuring marginal fit of zirconia crowns and indicated that using the external viewing is adequate and accurate for measuring the marginal fit without the need of destroying the specimens (Ortega et al., 2017).

**TABLE 3:** Summary of the articles included for final analysis

Study	Factors	Materials/system	SG	SS	Preparation design	Measure	Zr/Sinter	Results	G
Ahmed et al. (2019)	Sintering effect Different finish lines and crown thickness	Atlantis core file abutment fabrication	12	10	3 finish line 0.5, 1.0, 1.2 mm 2 crown thickness 0.8, 1.5 mm 12° taper	Vertical marginal gap Stereomicroscope ×40 then ImageJ software No cementation was performed.	IPS e.max ZirCAD LT 2 sintering protocols Standard 1450C for 9 h 50 min and fast 1520C for 2 h 50 min	1.0 mm finish line in both crown thicknesses showed the lowest VMG	H
Mejia, Yatani, Wakabayashi, and Nakamura (2019)	Different preparation taper Resin maxillary left central incisor	Digital impression then print out the resin die	7	10	‐8, −4, 0, 8, 12, 16, 22° tapers 50 μm cement space	Silicone replica technique No cementation was performed.	Kavo dental GmbH (3YTZP) Semi‐sintered zirconia	‐8° showed the highest marginal gap 58.2 μm and 22° showed the least marginal gap 42.1 μm	M
Khaledi, Vojdani, Farzin, Pirouzi, and Orandi (2019)	Sintering time Coping	Digital impression after scan spray 3D laser scanner (3ShapeD810; 3Shape, Copenhagen, Denmark)	3	10	7 mm high, 1 mm wide, 6° occlusal convergence angle, 90° Shoulder finish line 0.5 mm coping thickness	Digital microscope 18 measurements No cementation was performed.	1 h, 15 min for IPS e.max ZirCAD, 4 h 20 min for speed ZrO_2_, 7 h 20 min for conventional ZrO_2_	IPS e.max 41 μm Speed ZrO_2_ 43 μm Conventional 39 μm	M
Dahl, Dahl, and Ronold (2018)	Dual scan technique to measure the gap	Digital scanner trios	4	3	No details	Digitalized replica 24 marginal measurements/group No cementation was performed.	No sintering details HIP‐Zr (YTZP) Zir (YTZP)	Pre‐sintered Zr Fully sintered Zr Milled CoCr Laser sintered CoCr	L
Pilo, Folkman, Arieli, and Levartovsky (2018)	Cementation effect Cementation type	Conventional impression then Lava scanner RelyX U‐200 (RU200; 3 M ESPE, Seefeld, Germany), SmartCem 2 (SC2; Dentsply, Milford, DE, USA), G‐Cem automix (GCA; GC, Alsip, IL, USA), and Panavia 21 (PAN; Kuraray dental co ltd, Osaka, Japan)	2	10	0.4 mm chamfer finish line 6° taper, 50 μm cement thickness	Stereomicroscope ×50 20 measuring locations Absolute marginal gaps Sectioning	Lava frame blocks (3YTZP) No sintering details	Pre‐cementation 35 μm, post‐cementation 72 μm	M
Boitelle, Tapie, Mawussi, and Fromentin (2018)	Method of measurements compare 2D vs 3D (coping)	Conventional impression	2	30	Upper molar and premolar	Replica by silicone by light microscope vs triple scan by digital 3D map No cementation was performed.	No sintering details Zr material “nr”	Triple‐scan method was more reliable than silicone replica	M
Yus, Cantarell, and Alonso (2018)	Impression technique	Scanning silicone impression and scanning stone	2	30	Upper left molar Cr‐co 80 μm cement thickness	SEM × 600 12 points No cementation was performed.	Zirconium dioxide Zr material “nr”	Scanning silicone 22 μm Scanning stone 8.94 μm	M
Schriwer, Skjold, Gjerdet, and Oilo (2017)	Zirconia type	Soft milling vs hard milling	6	10	Upper premolar 0.5 mm chamfer finish line 9–12°	Replica No cementation was performed.	BruxZir 1530C Zirkonzahn1450‐1,555 Prettau 1,600 C NobelProcera N/A Denzir Y‐TZP Denzir AB 1800 C	Internal fit (IF) occlusal is larger and significant than axial IF	H
Ortega, Gonzalo, Gomez‐Polo, Lopez‐Suarez, and Suarez (2017)	Zirconia type	Procera, Lava, in‐ceram YZ, MC Cemented using conventional glass Ionomer cement (Ketac‐Cem Easymix, 3 M‐ESPE)	4	10	Steel spacemen Cement thickness 50 μm	SEM Measure external (EMG) and internal marginal gap (IMG), sectioning after cementation	3 zirconia groups and one MC group	Nobel Procera is the lowest (EMG = 39 μm and IMG = 41 μm) MC (EMG = 83 μm and IMG = 101 μm)	M
Kocaagaoglu, Kilinc, and Albayrak (2017)	Digital vs conventional impression Acrylic max premolar ➔ coping Die type: Stone	Convention impression (cn) then scanning ➔ in Eos X5 scanner. CEREC (Omnicam; Sirona)➔ group C (InLab SW 15.0; Sirona dental systems). 3Shape Trios‐3 ➔ group Tr (DWOS; dental wings).	3	10	Anatomic occlusal reduction 2 mm, 4–6° taper, axial reduction 1–1.5 mm, chamfer 1.0 mm, 0.5 mm above the CEJ, 0.5 mm copings with 30 *μm* cement space starting 1 mm from the margins	Replica 50 N then measured by stereomicroscopy ×50 21 measurements per coping: 8 for marginal, 8 for axial, 5 for occlusal gaps➔ 210 points No cementation was performed.	(ICE zircon translucent; Zirkonzahn SRL) Sintered at 1,500°C for 2 h with approximately 8°C/min heating and cooling rate	*MG: 85.6 μm for Cn, 58.7 μm for C, and 47.7 μm for Tr*. *AF: 85.4 μm for Cn, 76.1 μm for C, and 66.7 μm for Tr*. *OF: 177.3 μm for Cn, 177.9 μm for C, and 135.2 μm for Tr*.	H
Dahl, Ronold, and Dahl (2017)	Digital vs conventional impression Human mand first molar ➔ crowns	Trios scanner	2	18	No details	Triple scan protocol No cementation was performed.	(Zir; dental Direkt) No sintering details (HIP‐Zir; Denzir) Both 3YTZP	Zir 78 *μm* *HIP 81 μm* Lithium disilicate 76 *μm* M‐co‐Cr 90 *μm* L‐co‐Cr 82 *μm* Cast‐co‐Cr 58 *μm*	L
Pedroche et al. (2016)	Digital vs conventional impression (coping)	‐intraoral scanning (direct) ‐scanning of PVS (indirect) ‐ scanning of the gypsum cast/models (indirect)	3	10	Supragingival circumferential chamfer finish line, 2.0 mm occlusal reduction, 1.5 mm axial reduction, axial convergence angle of 6° and rounded angles	Replica 16 measurements Total 160 No cementation was performed.	(Metoxit, Thayngen, Switzerland) Zr material “nr”	MG Gypsum 87 *μm* PVS 71 *μm* Scanner 59.2 *μm*	M
Kale, Seker, Yilmaz, and Ozcelik (2016)	Effect of cement space on MG Ivorine right maxillary first molar	D9003Shape	3	5	0.5 mm axial reduction, chamfer finish line. Cement space 25 *μm* at the margins and at 1 mm above the finish lines was set at 30 *μm* for group 25–30, 40 *μm* for group 25–40, and 50 *μm f*or group 25–50	Stereoscopic zoom microscope 8 sites /crown A total of 120 measurements in the 3 groups No cementation was performed.	StarCeram Z‐nature; H.C. Starck)	Mean MG was 85 *μm* for group 25–30 68 *μm* for group 25–40 53 *μm* for group 25–50	M
Ha and Cho (2016)	2 CAD/CAM systems X veneering effect Monolithic crowns vs pressed veneered Zr copying Mand first molar acrylic	Zirkonzhan vs Ceramill	2	10	1 mm chamfer 2 mm occlusal reduction 5° convergence angle	Weight technique + Replica (figure pressure) then Leica microscope 5 points per replica Total 50 No cementation was performed.	Zirkonzhan: Sintered at 1,600°C for 10 h in a ZIRKONOFEN 600 furnace Ceramill: Sintered at 1,450°C for 11 h in a Ceramill Therm furnace	MG: Ceramill was between 106 and 117 μm, and the Zirkonzahn system was between 111 and 115 μm. IF: Ceramill was between 101 and 131 μm, and Zirkonzahn was between 116 and 131 μm	H
Dauti et al. (2016)	Digital vs conventional After cementation Left mand first molar	Lava C.O.S Zinc oxide phosphate cement (HOFFMANN'S CEMENT quick setting, Hoffmann dental Manufaktur, Berlin, Germany) was used.	4	20	0.8–1.2 mm chamfer F.L., 1.5 mm occlusal reduction, 6° convergence angle set to 0.01 mm thickness to a level of 1 mm above the margin and the cement space was set to 0.04 mm.	Cementation under constant finger pressure for 10–15 min, 292 measurements Half with stereomicroscope and half with SEM Sectioning	Zenostar Zr translucent blank a Cercon® heat plus furnace (DeguDent GmbH, Germany) for 8 h at 1,350°C (3YTZP) second generation zirconia	MG: Lava optical 96.283 μm, Conve optical 94.845 μm Lava SEM 99.265 μm, Conve SEM 83.376 μm AMG: Lava optical 191.543 μm, conv optical 158.609 μm Lava SEM 211.600 μm, Conve SEM 152.721 μm	M
Boitelle, Tapie, Mawussi, and Fromentin (2016)	Different CAD/CAM systems (copings) Acrylic model (a right max first molar and a left max first premolar)	(Cerec inLab system group) (dental wings/ Wieland Zenotec mini system group) (dental wings/Wieland Zenotec T1 system group)	3	20	A 1.5 mm a chamfer finish line, A 2 mm occlusal reduction For C, 20 μm margin 70 μm A and occ For Zm, Zt, 20 μm margin 70 μm A and 100 μm occ	3D triple‐scan optical technique More than 5,604 measurements No cementation was performed.	Pre‐sintered Zr Zr material “nr”	MG: C 54.32 μm, Zm 66.56 μm, ZT 61.08 μm AF: C 115.76 μm, Zm 100.01 μm, ZT 76.94 μm OF: C 143.82 μm, Zm 124.06 μm, ZT 127.41 μm	L
Vojdani, Safari, Mohaghegh, Pardis, and Mahdavi (2015)	Shoulder vs chamfer X firing porcelain effect (copings) Brass master dies	Conventional impression than scanned with a laser scanner (3Shape D810; 3Shape, Copenhagen K, Denmark)	4	10	A 1 mm chamfer and shoulder F.L., 6° occlusal convergence and a height of 7 mm. Anti‐rotational ledge. The copings were designed with a thickness of 0.5 mm considering the 30 μm spacer 1 mm short of margin.	(AMG) was taken at 18 points by use of a digital microscope and photographed sequentially at 230 × . No cementation was performed.	(VITA in‐ceram YZ‐14; Vident, Germany) (3YTZP) second generation zirconia	Chamfer coping 49.87 Chamfer crown 68.24 Shoulder coping 35.20 Shoulder crown 63.06	L
Torabi, Vojdani, Giti, Taghva, and Pardis (2015)	Different veneering techniques (layering (L), press‐over (P), and CAD‐on (C) techniques). Copings. A brass master die	Conventional impression than scanned with 3D‐laser scanner (3ShapeD810; 3Shape, Copenhagen K, Denmark)	3	10	7 mm height, 6° of occlusal convergence and a 90° shoulder of a 1 mm‐wide finish line. An anti‐rotational design in the axial surface.	(VMG) images from the 18 points using a digital microscope connected to PC and photographed at 230 × . No cementation was performed.	(IPS e.max ZirCAD, Ivoclar Vivadent)	Layering 63.06 Press‐over 50.64 CAD‐on 51.50	M
Pimenta, Frasca, Lopes, and Rivaldo (2015)	Different materials copings Model of left maxillary canine (acrylic resin) Marginal (MG) and internal fit (IF)	‐ zirconia YTZP (ZirkonZahn) *sintering for 7 h at 1,600°C in fire HTC Sirona ‐ lithium Disilicate LSZ (IPS e.max press system) 850°C ‐ nickel‐chromium NiCr alloy (lost‐wax casting) 400–800°C	3	5	Model of left maxillary canine (acrylic resin) Taper 6°, 2 mm incisal reduction, 1.2 mm facial reduction, rounded angles, 120° chamfer finish line➔ scanned and Zr master model was reproduced ➔ 15 stone die	Micro‐CT, Skyscan 1,173, 130 kV, 61 μA, 1 mm‐thick AL filter, a pixel size 9.91 μm, scanning time 90 min/specimen. Adobe Photoshop CTAn SkyScan software MG 4 points IF 9 points No cementation was performed.	Zirconia YTZP (ZirkonZahn) *sintering for 7 h at 1,600°C inFire HTC Sirona	MG.: YTZP 35.5 μm LSZ 76.19 μm NiCr 34.05 μm IF: YTZP 86.95 μm LSZ 73.36 μm NiCr 117.88 μm	M
Ortega, Gonzalo, Gomez‐Polo, and Suarez (2015)	3 CAD/CAM vs MCC maxillary first premolar	Metal‐ceramic Lava Procera Vita in‐ceram YZ	4	10	1 mm‐wide circumferential chamfer finish line and axial walls tapered at 6°.	All crowned were cemented with GI and sectioned BL applying a load of 10 N for 10 min then SEM	1) metal‐ceramic, (2) NobelProcera Zirconia, (3) Lava Zirconia, and (4) VITA In‐Ceram YZ.	MG: MCC 101.5 μm Lava 49.48 μm Procera 41.09 μm YZ 65.63 μm	L
Nakamura et al. (2015)	‐frame (coping) ‐crown (Zr‐veneer) ‐marginal, internal fit and fracture resistance	Hybrid Zr (fully sintered before milling) Dense Zr Commercial Zr Sintering 1,450°C for 2 h	3	7	Jacket crown epoxy➔ scanned ➔ milled to titanium abutment. Maxillary first molar Lingual collar for support Heavy chamfer 0.8 mm	Replica (9.6 N) MG: 40 points per specimen (microscope). IF: Fitting test material No cementation was performed.	Hybrid Zr Dense Zr Commercial Zr Sintering 1,450°C for 2 h	MG.: Frames: 48.9–58.2 μm Crowns: 48.6–59.4 μm IF: Frames: 125.6–139.5 μm Crowns: 128.2–138.2 μm	M
Lins, Bemfica, Queiroz, and Canabarro (2015)	3 fabrication systems 24 Zr copings Prefabricated titanium abutments	Ceramill Lava 3 M Neoshape Using 3 different laboratories YTZP 0.6 mm thickness	3	8	Cementation by using Zn phosphate cement 50 N UTM	Cement thickness after cementation, Zn phosphate then sectioning BL+ MD. Optical microscope ×100, ×200 6 locations	Y‐TZP	‐internal misfit were 72.1, 69.4, 76.4 μm ‐marginal discrepancy 40.9, 34.2, 39.3 μm ‐absolute marginal discrepancy 65.8, 70, 74.5 μm	M
Ji et al. (2015)	2 CAD/CAM systems Chamfer vs shoulder finish lines Maxillary first premolar	Prettau zirconia Zenostar ZR translucent Lithium disilicate IPS e.max press (control)	3	16	12° occlusal convergence angle, 1.5 mm occlusal reduction, a 1 mm shoulder (S) or deep chamfer (C)margin.	Crowns were bonded to stone dies with (rely X Unicem). Light microscope equipped with a digital camera (Leica DFC295) × 100 Sectioning	Prettau zirconia Zenostar ZR translucent Both are 5YTZP (third generation zirconia)	Prettau MG S 119 μm ZR MG S 92 μm Lithium disilicate MG S 41 μm Prettau MG C 109 μm ZR MG C 85 μm Lithium disilicate MG C 41 μm Prettau AMG S 74 μm ZR AMG S ‐14 μm Lithium disilicate AMG S 29 μm Prettau AMG C 38 μm ZR AMG C ‐52 μm Lithium disilicate AMG C 23 μm	M
Alghazzawi et al. (2015)	Different die 150 monolithic zirconia crowns Mandibular first molar melamine tooth.	Argen FZC Polyurethane master die 4 dies (3 stones and 1 Ti) 3shape scanner D9000	15	10	1 mm rounded shoulder OG 4 mm 12° total convergence 1.5 mm occlusal reduction Die spacer 35 μm	Replica technique 8 measurements Stereomicroscope ×40 No cementation was performed.	Argen (monolithic zirconia crown)	MG 49.32 to 91.20 mm.	M
Sener, Turker, Valandro, and Ozcan (2014)	Cementation effect (type of cement) Correlation to microleakage. Forty freshly extracted human first maxillary premolars	Correlation to microleakage	4	10	1 mm chamfer finish lines, 1.5 mm occlusal reductions, 6° of occlusal convergence	Replica ➔ optical microscope at (100X) (Leica). While 10 crowns were luted with MDP‐RC (Panavia) F 2.0, the other 10 were luted with GCI (Vivaglass) under a weight of 50 N for 10 min	Cercon system using a Cercon brain unit (DeguDent GmbH) The Precident DCS system (DCS dental AG)	Cercon (85 ± 11.4 μm) DC‐zircon (75.3 ± 13.2 μm) The mean cement thicknesses of GIC (81.7 ± 13.9 μm) and MDP‐RC (78.5 ± 12.5 μm)	VL
Re, Cerutti, Augusti, Cerutti, and Augusti (2014)	The effect of finish‐line configuration on zirconia coping 2 maxillary artificial teeth	Lava all‐ceramic system (3 M ESPE) Lava frame zirconia blanks, 3 M ESPE Lava furnace 200, 3 M ESPE	2	10	Axial reduction: 1–1.5 mm Occlusal reduction: 1.5–2 mm Round shoulder or chamfer Width 0.8 mm	100× optical microscope 50 measurements No cementation was performed.	Lava frame zirconia (3YTZP) first generation zirconia	VMG: Shoulder 30.2 ± 3 μm Chamfer 28.4 ± 4 μm	L
Miura, Inagaki, Kasahara, and Yoda (2014)	Different finish line widths Veneering effect Non‐anatomical crown	Not mentioned	3	15	Shoulder widths of 6, 8, and 1 mm	Replica No cementation was performed.	Cement thickness 30 μm Zr material “nr” 9 measurement per sample	No sig diff between before and after firing S 27 μm, RS0.2 30 μm, RS0.5 24 μm	L
Habib, Asiri, and Hefne (2014)	Different occlusal preparation Zirconia copings Extracted premolar	CAD4DENT CAD/CAM 3D digital scanner (7Series from dental wings Inc. Montreal, Canada).	3	15	(anatomical 30°, semi‐anatomical 15–30°, and non‐anatomical 0°), 2 mm occlusal reduction, 1 mm chamfer finish line, 1 mm axial reduction, 5–10° angle of convergence, cement space 0.01 mm	Copings were adjusted, cemented with conventional glass‐ionomer cement (Fuji‐ Cem; GC Int., Tokyo, Japan), and sectioned BL 9 measurements	Zr material “nr”	Overall mean gap values: 155.93 ± 33.98 μm Anatomical design had the best fit 139.23 ± 30.85 μm	M
Euan, Figueras‐Alvarez, Cabratosa‐Termes, and Oliver‐Parra (2014)	2 CAD/CAM systems X 2 finish lines Zr. Copings Extracted molar teeth	Lava all‐ceramic system and Lava chairside Oral scanner	4	10	Round shoulder (1 mm 90°) vs chamfer (1 mm 45°) 2 mm occlusal reduction, 6° axial convergence, 1–1.5 mm axial reduction.	Stereomicroscope coupled with digital camera. 20 measurements No cementation was performed.	Zirconia material and sintering protocol were not mentioned	C Lava all‐ceramic 64.07 μm C Lava oral 18.46 μm S Lava all‐ceramic 52.67 μm S Lava oral 14.99 μm	M
An, Kim, Choi, Lee, and Moon (2014)	Digital vs conventional Zr copings. Base‐metal dies from 1 maxillary central incisor	(iTrios)	3	10	2.0 mm incisal reduction, 1 mm axial reduction, 1.0 mm chamfer margin of 1.0 mm, 6° of convergence. A die spacer was applied to the stone dies of the CI group (60 mm) & simulated die spacers were set for the iP group and iNo group (60 mm), starting 1.0 mm from the margin	Replica + a light microscope at 50× magnification 4 locations No cementation was performed.	Zirblank; Acucera No sintering details	Conven better than digital CI group: 92.67 (13.94) mm; iP group, 103.05 (14.67) mm; and iNo group, 103.55 (16.50) mm.	L
Yildiz, Vanlioglu, Evren, Uludamar, and Ozkan (2013)	Two zirconia type Crowns (Zr copings + veneering)	Measurement before cementation	2	20	Chamfer Fl. Was 1 mm above the CEJ; preparation margins were not beveled. Core 0.5 mm thickness	Replicas using a light microscope (Leica at ×200). 40 measurements/specimen. 1,600 measurements for both zirconia systems	IPS ZirCAD zirconium oxide blocks (IZC) Lava zirconium oxide blocks (L)	MG was 89.26 μm for L crowns and 88.84 μm for IZC crowns, L crowns showed significantly larger axial and occlusal gaps than IZC crowns	L
Seelbach, Brueckel, and Wostmann (2013)	Simplified molar crown Accessible marginal inaccuracy IF: Internal fit	Lava, Cerec, iTero 1 step and 2 steps PVS impression	3	10	Circular chamfer	IF by 3D‐ coordinate at 50 points/crown. VMG. By traveling microscope & digital micrometer No cementation was performed.	IPS empress CAD, Ivoclar Vivadent, milled on CEREC Inlab	IF 49 ± 25 μm AMG 44 ± 26 μm	M
Sakrana (2013)	3 different zirconia materials Cementation effect Mandibular first premolar	In‐ceram zirconia Zirkonzhan Composite blocks	4	10	1 mm shoulder 2 mm occlusal reduction 6° taper 4 mm axial height	Steromicroscope Before and after cementation C‐gem self‐adhesive cement 12 measurements/ crown. 360 before and 360 after sectioning	No sintering details	Before cementation In‐ceram 56.3 μm Zirkonzhan 60.16 μm Composite 56.16 μm After cementation In‐ceram 84.2 μm Zirkozan 84.22 μm Composite 95.22 μm	L
Regish, Sharma, Prithviraj, Nair, and Raghavan (2013)	Zr vs NiCr copings X veneering effect Standardized metal master die prepared anterior crown	Ceramill (Amann Girrbach, Germany) Sintering for 8 h GI cement 5Kg	2	5	Chamfer finish line Triangular shaped orientation notch on the base	Cement thickness after sectioning with SEM.	Ceramill therm furnace for 8 h	NiCr was better than Zr but both deteriorated after veneering	L
Hamza et al. (2013)	2 fabrication systems X different materials Crowns Stainless steel dies Mandibular second molar	Cerc inLab vs Kavo Everst zirconia vs lithium disilicate	3	10	10.00 mm cervical diameter, 6.00 mm height, 6° total occlusal convergence. The occlusal surface was prepared with 2 sloping surfaces (one slightly beveled). Round shoulder 1.0 mm F.L.	Binocular microscope at ×100 8 predetermined measuring locations	No sintering details	VMG: Zr: 14–86 μm Lithium disilicate: 28–40 μm Lowest mean MG was Zr manufactured by Everest 14 ± 5.2 μm	M
Asavapanumas and Leevailoj (2013)	3 different finish line curvature × 3 diff materials Ivorine maxillary central incisor Then casted into cast in cobalt chromium molybdenum	3 diff finish line curvature 1, 3, 5 mm 3 diff materials cercon, IPS emax, Lava Using a polyether impression material	9	12	A 1.2 mm shoulder margin, 2 mm incisal reduction, 1.5 mm labial and axial reduction, and a total occlusal convergence of 6° 0.4 mm on a 30 μm die spacer	a stereomicroscope 4 sites No cementation was performed.	Cercon 0.4 IPS emax 0.6 (pressed) Lava 0.4	5 mm G (Cercon, 76.59 μm; IPS e.max, 106.44 μm; Lava, 128.34 μm) than for both the 3 mm G (Cercon, 60.18 μm; IPS e.max, 81.79 μm; Lava, 99.19 μm) &1 mm G (Cercon, 38.3 μm; IPS e.max, 52.22 μm; Lava, 69.99 μm)	M
Rinke, Fornefett, Gersdorff, Lange, and Roediger (2012)	2 different CAD/CAM Upper left second premolar acrylic tooth model Absolute marginal discrepancy (AMD)	Digitized with the Cercon EYE (EYE) scanner (DeguDent, Hanau, Germany), while the other 20 specimens per parameter were digitized using the 3Shape D‐700 scanner (3S) (DeguDent, Hanau, Germany).	2	10	A 1.0 mm, 360° rounded shoulder. The occlusal reduction was at least 1.5 mm, and the resulting convergence angle was set at 2 × 2° (4° taper). Cement space 60 μm	Light microscope Twenty‐four measurement points, Staggered by 15°,	Sintering for these specimens was done for 6 h at 1,350°C (Cercon heat, DeguDent, Hanau, Germany).	Maximum MG ranged from 112.24 ± 23.1 μm (EYE/COMP) to 144.6 ± 30.5 μm (EYE/EXPERT). Average MG ranged from 57.9 ± 6.49 μm (EYE/COMP) to 71.0 ± 10.8 μm (3S/COMP).	M
Euan, Figueras‐Alvarez, Cabratosa‐Termes, Brufau‐de Barbera, and Gomes‐Azevedo (2012)	Extracted molar Two different finish line configurations before and after porcelain firing cycles, after a glaze cycle, and after cementation Extracted molar	Lava™ system, veneer IPS e.max Ceram, cementation with RelyX™ Unicem, Aplicap™	8	10	Chamfer vs shoulder finish line Cementation	Measurements for MG using stereomicroscopy (40×) were performed at four stages: Copings (S1), after porcelain firing cycles (S2), after glazing (S3), and after cementation (S4)	No sintering details	Shoulder S1: 50.13 μm S2: 54.32 μm S3: 55.12 S4: 59.83 Chamfer S1: 63.56 S2: 71.85 S3: 74.12 S4: 76.97	M
Chandrashekar, Savadi, Dayalan, and Reddy (2012)	Maxillary central incisor Zr copings Compare between Zr vs NiCr marginal fit	Cercon for Zr➔ cement space 30 μm 1 mm away from the margin. Sintering 1,350°C for 6.5 hr. 0.5 mm thickness Lost wax technique for NiCr	2	15	Machined steel die 8 mm height 7 mm cervical dimeter 6° taper 1 mm shoulder finish line 90° Measurement mid b, mid L, mid M, mid D	SEM × 50 ImageJ software	1,350°C for 6.5 h	MG: Zr 39.32 μm MG: NiCr 129.98 μm	VL
Moldovan, Luthardt, Corcodel, and Rudolph (2011)	Copings internal fit accuracy, Cercon (dry‐mill) Vita in‐ceram (wet‐mill)	CAD/CAM wet and dry Zr copings 2 types of silicon	2	20	Rounded shoulder Made by reverse engineering	3D replica method (replica of cement space) by optical digitalization and computer‐assisted analysis 20,000 to 35,000 per die	According to Cercon® heat, Degudent GmbH, Hanau, Germany and Zyrkomat®, Vita Zahnfabrik, Bad Säckingen, Germany	Root means square Molars 28.6 (0.7) μm Premolars 24.9 (0.5) μm	M
Martinez‐Rus, Suarez, Rivera, and Pradies (2011)	Four different manufacturing system Ceramic copings Extracted mandibular first premolar AMD without cementation	In‐Ceram YZ (Cerec inLab system) A conventional waxing technique digitized by Cercon, and Procera zirconia (Nobel biocare AB 40 resin dies	4	10	A 1.2 mm deep chamfer 2 mm occlusal reduction Taper 6°	a stereomicroscope ×40 40 measuring points Marginal gap discrepancy No cementation was performed.	IZ N/A IY 1,530°C for 8 h CC 1,350°C for 6 h PZ 1,540°C	IZ: 29.98 μm (3.97) IY: 12.24 μm (3.08) CC: 13.15 μm (3.01) PZ: 8.67 μm (3.96)	M
Korkut, Cotert, and Kurtulmus (2011)	Cementation and aging Human premolars extracted Cementation with (Variolink II, Ivoclar‐ Vivadent).	(Procera all‐zircon, Cercon smart ceramics) in contrast to heat‐pressed ones (empress 2).	3	10	1 mm chamfer preparations 1 mm above the cemento‐enamel junction. 2 mm occlusal reduction 6° convergence angle was targeted to be 6°	Cemented using dual‐curing resin cement (Variolink II, Ivoclar‐Vivadent), then aging, then thermocycling. A computer‐aided stereomicroscope at 100× magnification (17 sites) after sectioning.	No sintering details	CAD/CAM (43.02 μm) Heat‐pressed (47.51 μm)	L
Grenade, Mainjot, and Vanheusden (2011)	Copings No veneering	Fabrication method Procera and Ceramill	2	10	In vivo prep	Cementation using self‐etch dual polymerized composite Resin cement (Clearfil esthetic cement; Kuraray medical Inc, Okayama, Japan) and sectioning 2 MG, 7 IG	Ceramill Therm according to manufacturer	Procera 51 Ceramill 81	M
Azar et al. (2011)	Preparation depth 0, 1.5, 3 mm Right max canine C Left mand first	Optical scanner (CEREC inLab, Sirona dental systems)	3	12	C0, C1.5, C3 P0, P1.5, P3	Cement space is 0 a light microscope No cementation was performed.	No sintering details	0➔BL 47 μm & MD 46 μm 1.5➔BL 58 μm & MD 43 μm 3➔BL 64 μm & MD 47 μm	M
Pak et al. (2010)	Two fabrication system X veneering Extracted maxillary central incisor	Digident and Lava	4	20	2–3 mm incisal reduction, axial reduction of approximately 1 mm, a 1 mm shoulder margin, 6 tapered angles, an approximate height of 7 mm	a light microscope with image processing at 50 points that were randomly selected No cementation was performed.	Pre‐sintered blanks Fully sintered blanks	Digident 61.52 μm before veneering and 83.15 μm after veneering. Lava 62.22 μm before veneering and 82.03 μm after veneering	M
Baig, Tan, and Nicholls (2010)	Different zirconia materials X different finish line Crowns	YTZP, pressed lithium disilicate and cast metal Shoulders or chamfers	6	10	1 mm shoulder, 20° taper 1.5 mm occlusal reduction 4 mm axial height	Stereomicroscope 6 measurement No cementation was performed.	No sintering details	CAD/CAM (66.4 μm) Heat‐pressed (36.6 μm) Cast metal (37.1 μm) No significant difference between shoulder and chamfer finish line	M

Abbreviation: SS, Sample size; SG, Study groups; Zr, zirconia; MG, marginal gap; AMG, absolute marginal gap; IF, internal fit; SEM, scanning electron microscope; MCC, metal ceramic crowns; CAD/CAM, computer‐aided design, computer‐aided manufacturing.

### 3.3 Description of the selected studies

Table 1 summarizes the articles included for final analysis.

## 4 DISCUSSION

The purpose of this systematic review is to map all the factors influencing the fit of zirconia crowns and/or copings and to update the latest review published in 2011 by Abduo et al. (2010). A clinically acceptable marginal gap for dental prostheses has been debated in the literature (Christensen, 1966; Fransson, Oilo, & Gjeitanger, 1985; McLean & Fraunhofer, 1971; Nawafleh, Mack, Evans, Mackay, & Hatamleh, 2013). The reported fit values for contemporary ceramic restorations in the literature range between 7.5 and 206 μm. In reviewing the literature, it was found that it is difficult to compare between marginal and internal adaptations of zirconia crowns between studies because of high variability of methodologies, including using different fabrication systems and impression techniques, different materials and different cements, different die materials and methods for assessing the fit. Other factors include the effects of different preparation designs, porcelain veneering and multiple porcelain firing, the effects of zirconia aging and thermocycling, and their correlation to microleakage. Only two studies have examined the effects of sintering technique or changing the sintering protocol on the adaptation and fit of zirconia crowns (Ahmed et al., 2019; Khaledi et al., 2019). A study (Ahmed et al., 2019) found a significant interaction between crown thickness, finish line width and sintering protocol on the marginal fit of zirconia crown; meanwhile, another study (Khaledi et al., 2019) did not find a significant difference among three different sintering times on the adaptation of zirconia copings.

Sixty percent of the reviewed studies scored “moderate” according to GRADE evaluation criteria. Evaluators were moderately confident in the effect estimate owing to the high variability of methodologies, absence of many important details, and/or quality of the peer‐reviewed journal. Seventy four percent of the studies were found to be missing the sintering protocol details, including the time/Temperature (t/T) ratio, as well as the technique used for sintering. Thirty percent of the studies investigated the effects of using different manufacturing systems (Euan et al., 2012; Ha & Cho, 2016; Lins et al., 2015), or zirconia types (Ortega et al., 2017; Schriwer et al., 2017) for fabricating zirconia prostheses or compared digital and conventional impression techniques using either a direct technique or replicas (Dahl et al., 2017; Ortega et al., 2017; Schriwer et al., 2017). Other studies investigated the effects of veneering within different manufacturing systems.

Ha and Cho (2016) evaluated the fit accuracy of two zirconia systems (Ceramill and Zirkonzhan) and studied the effect of pressed veneering over zirconia copies, comparing it to monolithic zirconia crowns. This was the only study to use the weight technique to determine the overall fit accuracy by weighing the silicone impression of the cement space. The marginal gap was smaller with Ceramill, and the internal gap was smaller with Zirkonzhan. The marginal and internal gaps were higher after veneering compared with before veneering. In this study, the authors used the manufacturer instructions to design and fabricate the prostheses, which may account for the differences in the weight techniques between the two systems.

Lins et al. (2015) compared the internal, marginal, and absolute marginal discrepancies of 24 zirconia copies fabricated by three CAD/CAM systems: Ceramill, Lava, and Neoshape, on Cone Morse (CM) abutment. No significant differences were found for the MG and AMG between the Ceramill and Lava (*p* = .147, .860), Ceramill and Neoshape (*p* = .878, .534), or Lava and Neoshape (*p* = .321, .842), respectively. The fact that the fabrication of the copings took place in three different laboratories according to the system used limits the validity of extrapolating the results to reflect the clinical conditions. The accuracy of data acquisition varied according to the system used and several optical impression technologies. In addition, the software used and the milling accuracy differed. However, the majority of crowns manufactured by the various ceramic systems satisfied the requirements for marginal adaptation.

Marginal and internal adaptations of zirconia were staggered and varied among the conventional and digital CAD/CAM ceramic systems. Multiple studies demonstrated superior (Kocaagaoglu et al., 2017; Pedroche et al., 2016) comparable (Dauti et al., 2016) or inferior (An et al., 2014; Dahl et al., 2017) marginal fit of digital impressions compared with conventional impressions. Consideration should be given to the study design, methodological parameters, and measurement tools when comparing the results, keeping in mind that increasing the processing steps in fabricating a restoration allows for the accumulation of errors. Therefore, direct digitalization would be anticipated to yield better marginal and internal fit of the restoration. Deformation of conventional impression materials would be expected while removing the impression from the prepared tooth and during casting procedures, in addition to expansion and shrinkage of the materials used.

Few studies have been reported investigating the effect of cement or cementation on the marginal gap. One study measured the absolute marginal gap of crowns cemented by four types of cement and was not able to find a linear correlation between microleakage and absolute marginal discrepancy (Cristian et al., 2016). A second study found that increasing the cement space increases the observed fit, and that group 25–50, which has a cement space of 25 μm at the margin and 50 μm starting 1 mm above the margin and elsewhere, had the smallest gap (53 μm) compared with groups 25–40 (68 μm) and 25–30 (85 μm), where marginal cement spaces was 25 μm and internal cement space was set to 40 and 30 μm starting 1 mm above the margin, respectively (Kale et al., 2016). This study should be interpreted with caution owing to the low number of measurement points investigated. Despite the fact that Cristian et al. (2016) measured the absolute marginal discrepancy in zirconia crowns cemented by four different types of cement, it would be more beneficial to measure the marginal gaps before and after cementation to test the effect of cementation on the marginal gap. The filler content and, consequently, the viscosity and flow can affect the crown seating and, therefore, the marginal gap. The effect of the force applied during cementation on the marginal gap is also important information.

One of the most influential factors for the marginal adaptation of zirconia prostheses is the preparation design. Habib et al. (2014) studied the effects of different occlusal surface preparations on the marginal and internal fits of zirconia copings on extracted premolars. The anatomical design (30°) had the best fit compared with the semi‐anatomical (15–30°) or non‐anatomical (0°) designs. Ahmed et al. (2019) investigated the effects of three finish line widths (0.5, 1.0, 1.2 mm) and two crown thicknesses (0.8, 1.5 mm) under the influence of two sintering protocols, and found that the 1.0 mm finish line showed the best results under both sintering protocols and for both crown thicknesses. Ji et al. (2015) found that the fabrication system and the finish line configuration significantly influenced the absolute marginal discrepancy (*p* < .05). In contrast to most studies, the study found that the chamfer finish line yielded a better marginal adaptation compared with the shoulder finish line. Vojdani et al. (2015) found that the marginal fit of shoulder copings was significantly better than that of chamfer copings; however, there was no significant difference between the two margins after firing the porcelain. Euan et al. (2012) and Euan et al. (2014) also found that shoulder finish line had better marginal fit compared to chamfer finish line in both systems. Euan et al. (2012) noticed that cementation procedures had no influence on the marginal gap in either group using the finger pressure technique. Although quantitative assessments could be performed between the chamfer and shoulder finish line studies, the authors are not confident of the results because of the variability in methodology between the studies.

Five studies evaluated the influence of veneering on the fit of zirconia restorations (Cho, Nagy, Goodman, Solomon, & Koike, 2012; Ha & Cho, 2016; Pak et al., 2010; Regish et al., 2013; Torabi et al., 2015). One study found a significant difference between the absolute marginal gaps of chamfer and shoulder finish line groups before (49.87, 35.20 μm) and after porcelain firing (68.24, 63.06 μm), and found that the absolute marginal fit of shoulder copings was significantly better than that of chamfer copings but with no significant difference between the two margins after firing the porcelain (Vojdani et al., 2015). The second demonstrated a significant effect of the veneering technique on the vertical marginal gap was demonstrated an increase in the vertical MG after porcelain veneering that was highest in the layering technique (63.06 μm) compared with the press‐on (50.64 μm) and CAD‐on (51.50 μm) techniques (Torabi et al., 2015). The third study compared marginal gaps between nickel chromium and zirconia copings before and after veneering and found that nickel chromium had a better marginal fit, but both deteriorated after porcelain veneering (Regish et al., 2013). The fourth study did not find an effect on marginal accuracy before or after veneering with the same system (Pak et al., 2010). However, the fifth study investigated the effect of multiple firing cycles on the marginal fit and found no significant difference in terms of ceramic type, finish line design, margin location, or their interactions (Cho et al., 2012).

Studies on veneering ceramic on zirconia frameworks showed a multifactorial effect. The difference noticed between the studies could be related to the technique of veneering, margin design, porcelain firing, and the thickness, materials, and design of the veneering layer and the zirconia framework. The veneering effect could be explained by the fact that porcelain veneering involves heating the ceramic to its melting point. When the porcelain particles melt, they gather to fill the voids and induce a compression stress that leads to coping deformation around the circumference of the margin. In addition, the difference in mismatch of coefficient of thermal expansion between the veneering layer and the zirconia place the veneering ceramic under compression stresses, which, in turn, lead to margin distortion and, consequently, may deteriorate the fit.

Generally, there was no conclusive evidence on the best methodology to evaluate the fitting accuracy of zirconia crowns. Most of the studies have assessed the marginal fit of zirconia prostheses with the direct view technique using external microscopes and the internal fit with either internal microscopes (sectioning after cementation) or the replica technique. Assessment of internal fit using microscopes is a destructive method that requires sectioning of the specimen. One of our selected studies evaluated the marginal and internal fits of zirconia crowns using micro‐CT; meanwhile two studies used the triple scan technique, one used CNC‐Rapid profilometry, and one used the weight technique. Therefore, direct comparisons between studies were not possible, as studies varied in their methods of measurements as well as other factors previously mentioned.

According to the previous studies, there was no consensus on how many measurements should be taken per specimen to obtain an accurate and clinically relevant conclusion on marginal and internal gaps. In addition, our findings highlighted the importance of obtaining individual measurements without combining the results, which complicates interpretation of the results in terms of their clinical relevancy. Groten, Axmann, Probster, and Weber (2000) suggested that 50 measurements along the crown margin can provide clinically relevant information if the measurements are taken at equal distances or are randomly selected. In contrast, Gassino, Monfrin, Scanu, Spina, and Preti (2004) reported that the minimum number of measurements required to ensure clinical relevancy for gap measurements is 18.

None of the studies evaluated the effect of aging on the marginal and internal adaptation of zirconia restoration. Few studies have attempted to evaluate the effects of sintering parameters on the mechanical properties of zirconia or to compare conventional sintering techniques (Almazdi, Khajah, Monaco Jr., & Kim, 2012; Ersoy, Aydoğdu, Değirmenci, Çökük, & Sevimay, 2015; Kim, Ahn, Kim, Kim, & Kim, 2013; Marinis et al., 2013; Sulaiman et al., 2015) and only two have investigated the effects of using different sintering protocols on marginal fitting (Ahmed et al., 2019; Khaledi et al., 2019).

One of the limitations of this systematic review is that it relied on two databases for the identification of potentially eligible studies. Future studies may investigate the effect of other sintering protocols used for zirconia restorations and their impact on marginal adaptation. Studies with long‐term follow‐up of the clinical performance of monolithic zirconia crowns are needed.

## 5 CONCLUSIONS

Within the limitation of this systematic review, we can conclude the following:

1. Regarding the effect of preparation design on the marginal fit of zirconia crowns, shoulder finish lines had a slightly better marginal adaptation compared with chamfer finish lines. Crowns obtained from anatomical tooth preparation had better marginal and internal fit than semi‐anatomical or non‐anatomical designs. Furthermore, increasing the cement space significantly improved the fit of zirconia crowns.
2. Zirconia crowns obtained from digital impression techniques had comparable results to those of conventional impression techniques. Recent studies have showed the superiority of zirconia adaptation on crowns obtained from digital impressions.
3. Most of the studies reviewed investigated the fit of the 3 mol% yttria tetragonal polycrystalline zirconia materials (YTZP) (first‐ and second‐generation zirconia), whereas few studies investigated the fit of the third‐generation zirconia 4 and 5 mol% yttria partially stabilized zirconia. Meanwhile, many studies failed to specify the type of zirconia material used in their study, and none of the reviewed studies investigated the fit of nano‐structured zirconia materials.
4. Veneering porcelain on zirconia copings increased the marginal gap compared with that without veneering. Layering technique showed higher marginal gaps compared with press and CAD‐on techniques.
5. Regardless of the type of cement use, cementation increased the marginal gap of zirconia crowns. Further studies should focus on the effect of cement thickness and load applied during cementation on the marginal adaptation of zirconia crowns.

### Clinical Significance

Precise marginal fit is an essential component for the clinical success of dental restorations. Misfit of the prosthesis margin generates a potential space between the restored and prepared tooth; this gap can accumulate bacterial plaque and consequently jeopardize the longevity of the treatment. Therefore, understanding the factors influencing the marginal and internal adaptability of zirconia prostheses will assist in the improved preparation, designing, and processing zirconia prostheses for achieving clinical success.

## CONFLICTS OF INTEREST

The authors have no conflicts of interest to declare.
